# Differential prognosis of metastatic colorectal cancer patients post-progression to first-line triplet chemotherapy plus bevacizumab, FIr-B/FOx, according to second-line treatment and *KRAS* genotype

**DOI:** 10.3892/ijo.2013.2179

**Published:** 2013-11-15

**Authors:** GEMMA BRUERA, KATIA CANNITA, ALDO VICTOR GIORDANO, ROBERTO VICENTINI, CORRADO FICORELLA, ENRICO RICEVUTO

**Affiliations:** 1 Medical Oncology, University of L’Aquila, I-67100 L’Aquila, Italy; 2 Radiology, University of L’Aquila, I-67100 L’Aquila, Italy; 3 Hepatobiliary-Pancreatic Surgery, S. Salvatore Hospital, University of L’Aquila, I-67100 L’Aquila, Italy; 4 Department of Biotechnological and Applied Clinical Sciences, University of L’Aquila, I-67100 L’Aquila, Italy

**Keywords:** metastatic colorectal cancer, triplet chemotherapy plus bevacizumab, *KRAS* genotype, re-challenge, c.35 G>A *KRAS* mutation, post-progression

## Abstract

Clinical outcome post-progression to first-line triplet chemotherapy (CT) plus bevacizumab (FIr-B/FOx) was evaluated in metastatic colorectal cancer (MCRC) patients (pts). Second-line treatment was selected according to fitness, *KRAS* genotype, previous efficacy and safety. Efficacy was evaluated and compared according to treatment or *KRAS* genotype, using log-rank analysis. Among 54 pts, median overall survival (OS) post-progression was 12 months, significantly better in 40 (74.1%) treated compared to 14 (25.9%) who died without further treatment. Second-line surgical treatment, 4 pts (7.4%), medical treatment, 36 pts (66.7%): triplet CT plus targeted agent, 10 (18.5%); triplet regimens, 19 (35.2%); doublet/monotherapy, 7 (13%). At follow-up of 14 months, objective response rate (ORR) was 38%, metastasectomies 12.5%, progression-free survival (PFS) 10 months, OS 14 months. According to treatment, ORR, metastasectomies, PFS and OS were significantly favourable in triplet CT plus targeted agent compared to triplet, respectively: 80%, 40%, 13 months, not reached; 28%, 6%, 8 months, 11 months. PFS and OS were significantly worse in c.35 G>A mutant compared to wild-type and/or other mutant patients. Prognosis after progression to first-line FIr-B/FOx may be significantly favourable in MCRC pts re-challenged with intensive regimens, and unfavourable in c.35 G>A *KRAS* mutant patients.

## Introduction

Clinical management of MCRC is faced with different options and lines of treatment strategies according to the fitness of the patients, extension of metastatic disease and *KRAS* genotype (1–5). First line triplet regimens significantly increased PFS up to 7.2–10.6 months and OS up to 19.9–26.1 months over doublet regimens, also integrated with secondary resection of liver metastases in liver-limited (L-L) disease (2,4,6). After progression to first line treatment strategy, 50–80% MCRC pts receive a second line treatment (4,7–11). Randomized clinical trials and pooled analysis demonstrated that exposure of patients to all three most active chemotherapeutic drugs is associated with the longest OS and similar efficacy (7), regardless of the sequence of administration. OS after progression does not correlate with any second line treatment (8).

Second line irinotecan (CPT-11), in 5-fluorouracil (5-FU) refractory MCRC pts, achieved median PFS of 3–4 months and OS 9.9 months (12,13). Doublet FOLFOX6 or FOLFIRI showed similar efficacy (7), with <2% metastasectomies. FOLFOX4 compared to CPT-11 significantly achieved ORR 28% and PFS 6.2 months, with no difference in OS (14). The addition of oxaliplatin (OXP) to CPT-11 showed significantly increased ORR 22%, PFS 5.3 months and median OS of 13.4 months (15). The addition of bevacizumab (BEV) to FOLFOX4 significantly increased ORR to 22.7%, median PFS 7.3 months, and median OS 12.9 months (16). Among pts treated with first line triplet FOLFOXIRI chemotherapy, ORR was 23%, PFS 5.9 months, OS 13.2 months (10). In EGFR-overexpressing MCRC pts previously treated with 5-FU, CPT-11 and OXP, cetuximab significantly improved ORR, PFS and OS, compared to best supportive care (BSC) (17,18). In CPT-11 or 5-FU/OXP refractory pts, cetuximab addition to CPT-11 showed significantly higher ORR of 22.9% and 16.4%, PFS 4.1 and 4.0 months, respectively (19,20). A significant interaction was demonstrated between *KRAS* wild-type genotype and effectiveness of cetuximab compared to BSC alone, increasing PFS up to 3.7 months and OS up to 9.5 months (21). Panitumumab confirmed the significantly positive predictive effect of *KRAS* wild-type status, with ORR of 17%, median PFS 12.3 weeks, median OS 8.1 weeks, compared to mutant genotype (22,23). In *KRAS* wild-type pts, the addition of panitumumab to FOLFIRI significantly increased ORR of 35% and PFS 5.9 months, with a trend toward increased OS (24).

More intensive first line medical treatment consisting of triplet chemotherapy plus targeted agent can increase activity, thus increasing resection rate of liver metastases and clinical outcome of MCRC pts (1,2,6,25,26). We recently proposed a phase II study of BEV addition to triplet chemotherapy, according to FIr-B/FOx schedule (1) reaching ORR of 82%, 54% liver metastasectomies in L-L disease, median PFS 12 months, median OS 28 months (1,3). *KRAS* wild-type pts with L-L disease may achieve significantly greater benefit from integration with liver metastasectomies compared to other/multiple metastatic (O/MM) pts, with respect to *KRAS* mutant pts (3,5).

The present study evaluated clinical outcome of the fit MCRC pts after progression to FIr-B/FOx and, retrospectively, the prognostic relevance of second line treatments and *KRAS* genotype.

## Materials and methods

### Patient eligibility

Sixty-seven fit MCRC pts were enrolled in previously reported phase II study (1) and in the expanded clinical program proposing FIr-B/FOx association as first line treatment. Pts had histologically confirmed diagnosis of measurable MCRC, age 18–75 years, World Health Organization (WHO) performance status ≤2, adequate hematological, renal and hepatic functions, life expectancy >3 months. The study was approved by the Local Ethics Committee (Comitato Etico, Azienda Sanitaria Locale n.4 L’Aquila, Regione Abruzzo, Italy) and conducted in accordance with the Declaration of Helsinki. All patients provided written, informed consent. After progression, second-line treatment was selected among medical and/or surgical options available in clinical practice, according to age (< or ≥75 years), patient fitness (performance status, Comorbidity Index Rating Scale), safety of FIr-B/FOx treatment, activity and efficacy of first line treatment [objective response (OR), PFS], *KRAS* genotype. Pts with performance status 3 were not treated, nor pts with clinical complete response (cCR) until progression.

### Medical treatment regimens

Medical treatments included: rechallenge of FIr-B/FOx or triplet chemotherapy plus cetuximab; triplet, doublet or mono-chemotherapy regimens. FIr-B/FOx schedule consisted of weekly timed-flat-infusion 5-FU (TFI 5-FU), associated to weekly alternating CPT-11/BEV or L-OXP (1): TFI/5-FU (Fluorouracil Teva; Teva Italia, Milan, Italy), 900 mg/m^2^/die, over 12 h (from 10:00 p.m. to 10:00 a.m.), on days 1–2, 8–9, 15–16 and 22–23; CPT-11 (Campto; Pfizer, Latina, Italy), 160 mg/m^2^, days 1 and 15; BEV (Avastin; Roche, Welwyn Garden City, UK), 5 mg/kg, days 1 and 15; l-OXP (Eloxatin; Sanofi-Aventis, Milan, Italy), 80 mg/m^2^, days 8 and 22; cycles every 4 weeks. Triplet chemotherapy plus cetuximab consisted of: TFI/5-FU, 800 mg/m^2^/die, days 1–2, 8–9, 15–16 and 22–23; CPT-11, 140 mg/m^2^, days 1 and 15; l-OXP, 80 mg/m^2^, days 8 and 22; cetuximab (Erbitux; Merck, Darmstadt, Germany), 400 mg/m^2^ initial dose, then 250 mg/m^2^/week; cycles every 4 weeks. Triplet FIr/FOx regimen, doublets and mono regimens were administered according to previously reported schedules (27,28).

### Study design

Pts were assessed at the time of progression to first line treatment and every 2–3 cycles of second line treatment. A multidisciplinary team, consisting of a medical oncologist, liver surgeon, radiologist, evaluated resectability, according to previously reported resectability categories (3). Clinical criteria of activity and efficacy were: ORR, resection rate of metastases, PFS, OS. ORR was evaluated according to RECIST criteria (29); pathologic complete response was defined as absence of residual cancer cells in surgically resected specimens. Clinical evaluation of response was made by CT-scan; PET was added based on investigator assessment. Liver metastasectomies were defined as: R0, if radical surgery; R1, if radiofrequency was added. Surgery was recommended >4 weeks after BEV discontinuation. PFS and OS were evaluated using the Kaplan-Meier method (30). PFS was defined, as the length of time from the beginning of treatment and disease progression or death (resulting from any cause) or to the last contact; OS as length of time between beginning of treatment and death or to last contact. Prognostic relevance of second line treatments and of *KRAS* genotype was retrospectively assessed, using log-rank test to compare PFS and OS (31).

### Mutational analysis

*KRAS* and *BRAF* genetic analyses were performed on paraffin-embedded tissue blocks from primary tumor and/or metastases, through selection of tumor cells, and DNA extraction, as previously described (5). Genotype status was assessed for *KRAS* codon 12–13 and *BRAF* c.1799 T>A (V600E) mutations by SNaPshot^®^ multiplex screening for *KRAS* mutations and *KRAS/BRAF* mutations in 36 and 32 samples, respectively (32,33); direct sequencing was performed to detect *KRAS* mutations in 26 samples. SNaPshot multiplex assay was performed as reported (32,33). Briefly, *KRAS* exon 2 and BRAF exon 15 were simultaneously PCR-amplified using specific primers and analyzed using the ABI PRISM SNaPshot Multiplex kit (Applied Biosystems, Foster City, CA, USA) with five primers including at their 5′-end an additional tail allowing their simultaneous detection. Sense primers allowing the extension at nucleotides *KRAS* c.34G, c.35G, c.37G, c.38G and *BRAF* c.1799T were used and multiplex SNaPshot reaction was performed as reported (32). *KRAS* exon 2 sequence was performed from PCR-amplified tumor DNA using the Big Dye V3.1 Terminator kit (Applied Biosystems), electrophoresis in ABI PRISM 3130xl Genetic Analyzer (Applied Biosystems), and analysis using the GeneMapper Analysis Software version 4.0 (Applied Biosystems).

## Results

### Patient demographics

Fifty-four MCRC pts developed disease progression (80.6%), among 67 consecutively treated with first-line FIr-B/FOx regimen. Fourteen pts (25.9%) died without further treatment. Forty pts (74.1%) underwent second line treatment, 4 pts surgical (7.4%), 36 pts medical (66.7%) (Table I). Second line medical treatment were: triplet chemotherapy plus targeted agent, 10 pts (18.5%); triplet regimens, 19 pts (35.2%); doublet regimen, 3 pts (5.6%); mono-therapy, 4 pts (7.4%). Among 51 *KRAS* evaluated pts (94.4%), 26 wild-type and 25 (49%) mutant, second line treated were 21 (80.8%) and 17 (68%), respectively; death events without further treatment were 5 (19.2%) and 8 (32%), respectively. Cetuximab-containing regimen was also administered in 4 EGFR-overexpressing/*KRAS* mutant pts, before recommendation of anti-EGFR treatment in *KRAS* wild-type patients.

Table II describes features of the 40 treated pts: male/female ratio, 26/14; median age, 65 years; young-elderly pts (≥65/<75 years), 18 (45%); metastatic disease metachronous 37.5%, synchronous 62.5%. Metastatic sites: liver 22 pts 55%), lung 17 pts (42.5%), lymph nodes 17 pts (42.5%); local recurrence 13 pts (32.5%). Metastatic site was single in 16 pts 40%), multiple in 24 pts (60%). Single metastatic sites were: liver 9 pts (22.5%), other than liver 7 pts (17.5%). Liver metastases were single in 3 pts (7.5%) and multiple in 20 pts (50%). The features of the patients who died without further treatment were not different from the treated patients. Among 38 second line treated MCRC pts evaluated for *KRAS* genotype, 21 wild-type (55.3%) and 17 mutant (44.7%), demographic and baseline features were, respectively: male/female ratio, 17/4 and 8/9; metachronous/synchronous metastatic disease, 10/11 (48/52%) and 5/12 (29/71%) pts. Distribution according to extension of metastatic disease, L-L and O/MM, was, respectively: *KRAS* wild-type, 3 (14%) and 18 (86%); *KRAS* mutant, 6 (35%) and 11 (65%). *KRAS* mutations detected in 17 pts were: codon 12, 14 pts (82.3%), specifically c.35 G>A 8 pts (47.7%), c.35 G>T 5 pts (29.4%), c.35 G>C, 1 patient; codon 13, 3 pts (17.6%), c.38 G>A 2 pts (11.7%) and c.37_39 dupl, 1 patient. Twenty-three tumoral samples (62.2%) were analysed for *BRAF* and no *BRAF* mutation was detected; 13 out of 21 *KRAS* wild-type MCRC pts were *KRAS* and *BRAF* wild-type.

### Activity and efficacy

**Figure 1.**
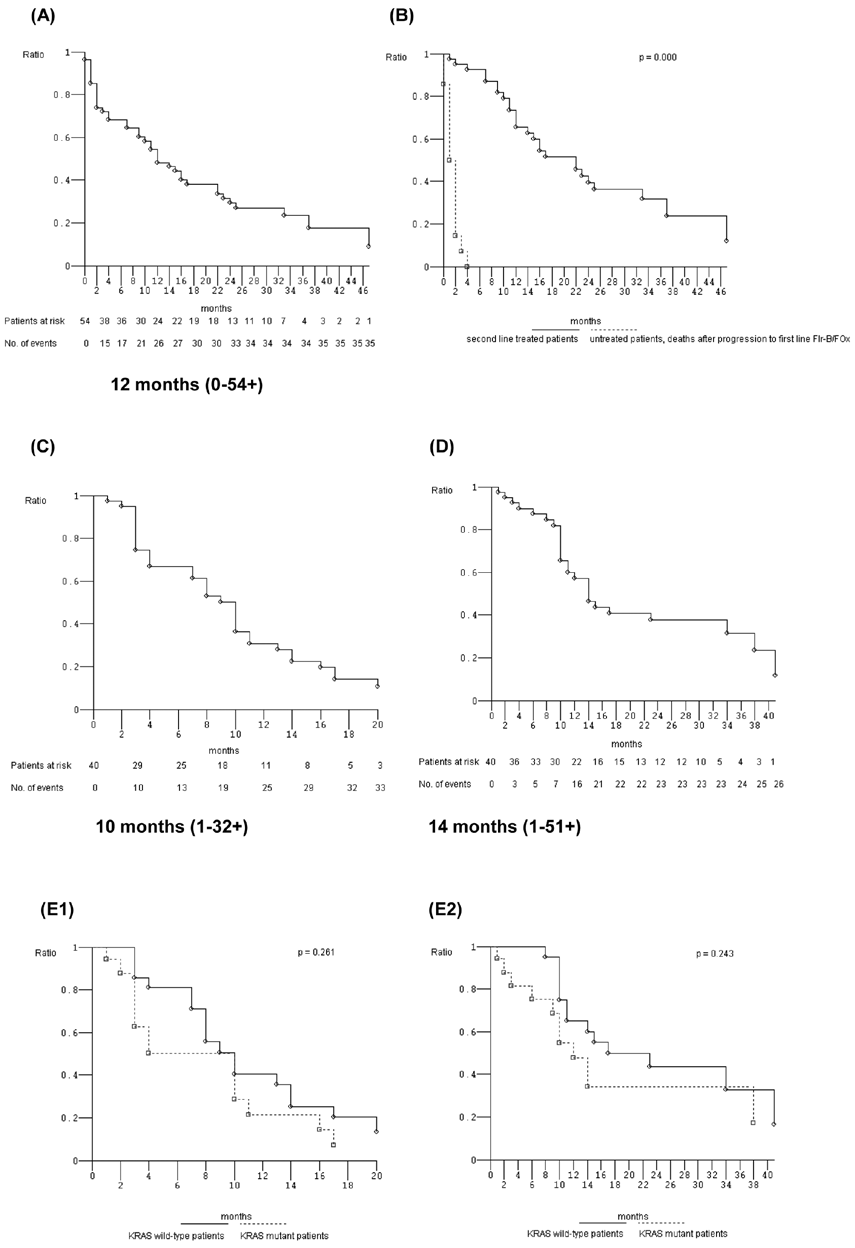
Kaplan-Meier survival estimate. (A) Post-progression from first line FIr-B/FOx regimen overall survival; (B) Post-progression from first line FIr-B/FOx regimen overall survival, second line treated patients versus untreated patients; (C) Second line treatment, overall patients, progression-free survival; (D) Second line treatment, overall patients, overall survival; (E) Second line treatment, *KRAS* wild-type versus *KRAS* mutant patients: (E1) Progression-free survival; (E2) Overall survival.

At a median follow-up of 11.5 months, overall OS post-progression to FIr-B/FOx was 12 months (0–54+ months) (Fig. 1A). Among the 40 pts who received second line treatment and the 14 untreated pts, median OS after progression was significantly different: 22 months (1+−54+) and 2 months (0–4 months), respectively (Fig. 1B). Intent-to-treat analysis of 34 evaluable pts (Table IIIA) showed ORR 38% (α 0.05, CI ± 17). We observed 13 objective responses: 10 partial responses (29%) and 3 complete responses (CR 9%); 10 were stable disease (29%); and 11 progressive disease (32%). Disease control rate was 68% (α 0.05, CI ± 16). At median follow-up of 14 months, median PFS was 10 months (1–32+): 33 events occurred (Fig. 1C). Median OS was 14 months (1–51+ months): 26 events occurred (Fig. 1D). Secondary metastasectomies were performed in 5 pts (12.5%): 2 liver resections, 2 peritonectomies, 1 lymph node resection. Two liver metastasectomies (R0) were performed out of 22 pts with liver metastases (9%), and out of 9 pts with L-L disease (22%), without surgery-related complications. A pathologic CR was obtained after 3 cycles of FIr-B/FOx rechallenge inducing a cCR in a c.35 G>T *KRAS* mutant patient with multiple liver-only metastases. Among 18 evaluable *KRAS* wild-type pts, ORR was 50% (CI ± 24) (Table IIIA). We observed 9 objective responses: 7 partial responses (39%) and 2 CR (11%); 5 stable diseases (28%); 3 progressive diseases (17%). Disease control rate was 82% (CI ± 19). Metastasectomies were performed in 3 pts (15%). Median PFS was 10 months (3–31+ months), 17 events occurred. Median OS was 17 months (5+−51+ months), 13 events occurred. Among 14 evaluable *KRAS* mutant pts, ORR was 29% (CI ± 26) (Table IIIA). There were 4 objective responses: 3 partial responses (21%) and 1 CR (7%); 3 stable diseases (21%); 7 progressive diseases (50%). Disease control rate was 50% (CI ± 27). Metastasectomies were performed in 2 pts (12%). Median PFS was 10 months (1–32+ months), 14 events occurred. Median OS was 12 months (1–39+ months), 11 events occurred. *KRAS* wild-type compared with mutant pts did not show significantly different PFS nor OS (Fig. 1E1 and E2).

### Prognostic relevance of second line treatments and of c.35 G>A KRAS mutation

**Figure 2.**
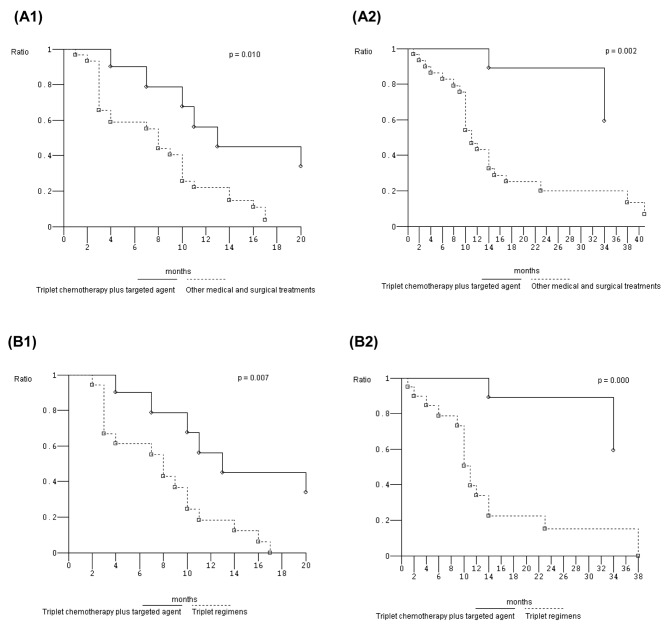
Kaplan-Meier survival estimate. (A) Second line treatment, triplet chemotherapy plus targeted agent versus other medical and surgical treatments. (B) Second line treatment, triplet chemotherapy plus targeted agent versus triplet regimens. (1) Progression-free survival; (2) Overall survival.

Among 10 pts treated with triplet chemo-therapy plus targeted agent (Table IIIB), ORR was 80% (α 0.05, CI ± 26). We observed 8 objective responses: 5 partial responses (50%) and 3 CR (30%); 1 stable disease (10%); 1 progressive disease (10%). Median PFS was 13 months (4–32+): 6 events occurred. Median OS was not reached (6+−39+ months), at median follow-up of 31.5 months; 2 events occurred for a 2-year OS 80%. Secondary metastasectomies were performed in 4 pts (40%). Among 19 pts treated with triplet regimens (Table IIIB), ORR was 28% (α 0.05, CI ± 21). We observed 5 partial responses (28%); 6 stable diseases (33%); 7 progressive diseases (39%). Median PFS was 8 months (1+−17): 17 events occurred. Median OS was 11 months (1+−38 months): 16 events occurred. Among 7 pts treated with doublet or mono-regimens, we observed 6 progressive diseases (86%), median PFS 4 months (1–17 months), median OS 10 months (1–17 months). Among 4 pts who underwent surgery as second line treatment, median PFS was 14 months (3–14); median OS 41 months (10–42+ months). Eighteen pts (45%) received a third line treatment. PFS and OS were significantly different in pts treated with triplet chemotherapy plus targeted agent compared to other second line treatments (p=0.010 and 0.002, respectively), and to triplet regimens (p=0.007 and 0.000, respectively) (Fig. 2).

**Figure 3.**
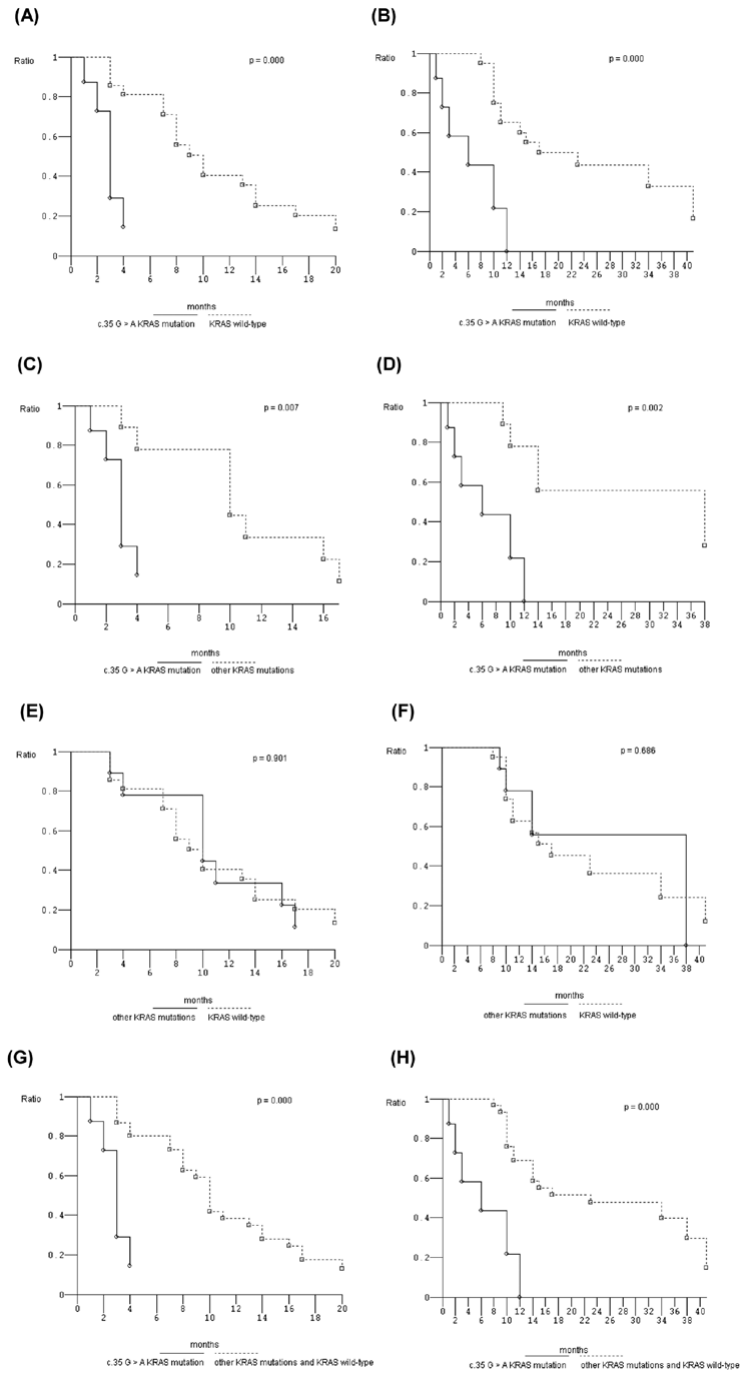
Kaplan-Meier survival estimate. (A) Progression-free survival of c.35 G>A *KRAS* mutant patients versus *KRAS* wild-type patients; (B) Overall survival of c.35 G>A *KRAS* mutant patients versus *KRAS* wild-type patients; (C) Progression-free survival c.35 G>A *KRAS* mutant patients versus other *KRAS* mutant patients; (D) Overall survival c.35 G>A *KRAS* mutant patients versus other *KRAS* mutant patients; (E) Progression-free survival, other *KRAS* mutant patients versus *KRAS* wild-type patients; (F) Overall survival, other *KRAS* mutant patients versus *KRAS* wild-type patients; (G) Progression-free survival, c.35 G>A *KRAS* mutant patients versus other *KRAS* mutant plus *KRAS* wild-type patients; (H) Overall survival, c.35 G>A *KRAS* mutant patients versus other *KRAS* mutant plus *KRAS* wild-type patients.

Retrospective analysis of clinical outcome in c.35 G>A *KRAS* mutant pts showed significantly worse PFS and OS compared to *KRAS* wild-type pts (p=0.000, and 0.000, respectively) (Fig. 3A and B), and to other than c.35 G>A *KRAS* mutant pts (p=0.007, and 0.002, respectively) (Fig. 3C and D). No different clinical outcomes were reported in other than c.35 G>A *KRAS* mutant compared to wild-type pts (Fig. 3E and F). PFS and OS were also significantly worse in c.35 G>A *KRAS* mutant pts compared to other than c.35 G>A *KRAS* mutant plus *KRAS* wild-type pts (Fig. 3G and H).

## Discussion

Among fit MCRC pts treated with first line FIr-B/FOx regimen, adding BEV to triplet chemotherapy, 74.1% underwent a second line treatment, in the range of reported 50–80% (7–11); 25.9% died without receiving further antitumoral treatment. Median OS post-progression to FIr-B/FOx was 12 months, including untreated pts and significantly better in second line treated patients. At median follow-up of 14 months, the 34 evaluable pts treated with re-challenge of triplet chemotherapy plus targeted agent (18.5%), triplet (35.2%) or less intensive regimens (13%), reported an overall ORR of 38%, median PFS 10 months, median OS 14 months. Secondary metastasectomies were performed in 12.5% (22% of L-L disease), all previously challenged with first-line FIr-B/FOX regimen and secondary surgery. Doublet FOLFOX4 schedule, or OXP associated to CPT-11 reported significantly increased ORR of 22 and 28%, and PFS 6.2 and 5.3 months, compared to CPT-11 alone, respectively. Median OS was 13 months, significantly increased only with OXP/CPT-11 regimen (14,15). Randomized studies of cetuximab plus CPT-11 in EGFR-overexpressing pts, previously treated with CPT-11 or with 5-FU/OXP, respectively showed significantly improved ORR of 16.4 and 22.9% and PFS 4 months (12,20). Triplet FOLFOX4-BEV association, after progression to 5-FU/CPT-11, demonstrated significantly increased ORR 22.7%, median PFS 7.3 months, and median OS 12.9 months (16). Recently, FOLFIRI-aflibercept, after progression to OXP-containing chemotherapy, gained significantly increased median OS 13.5 months (34). A randomized trial reported that BEV associated with 5-FU-based chemotherapy, after first line BEV-containing regimen, significantly improved clinical outcome (35). In *KRAS* wild-type pts, triplet panitumumab/FOLFIRI regimen reported significantly increased ORR of 35% and median PFS 5.9 months (23,24). Thus, OS after progression does not correlate with any second line treatment (8) in clinical trials and few secondary resections of metastases were reported after second line treatment (7).

Retrospective analysis of 32 pts (24%) achieving OR and progressed >3 months, who were re-challenged with FOLFOXIRI, reported significantly longer PFS (8.2 months) and OS (19.3 months), with respect to doublet regimens (10,11). In our present analysis, second line triplet regimens, proposed to 19 pts (47.5%) achieved ORR 28%, secondary metastasectomies 6%, median PFS 8 months, median OS 11 months. Re-challenge of triplet chemotherapy associated to targeted agent, proposed to 10 pts (25%), with previous OR, long PFS (≥10 months), off-treatment interval ≥3 months and no previous limiting toxicities, achieved ORR 80%, that correlated with 40% secondary surgical resections, median PFS 13 months, and 2-year OS 80% (median OS not reached at median follow-up 31.5 months). PFS and OS were significantly favourable in pts treated with triplet chemotherapy plus targeted agent compared to triplet regimens. Present data confirm that re-challenge of intensive medical treatment is feasible in a selected subgroup of MCRC pts, with high activity, efficacy and effectiveness of secondary metastasectomies. Prospective studies will address if medical and surgical re-challenge can be the standard multidisciplinary second line strategy.

Direct comparison of PFS and OS in *KRAS* wild-type compared to mutant pts failed to significantly differentiate prognosis in second line, as it was previously reported in first line treated MCRC pts (5,36,37). In *KRAS* mutant pts harbouring the prevalent c.35 G>A transversion, median PFS and OS were significantly worse compared to *KRAS* wild-type pts and/or other than c.35 G>A *KRAS* mutant pts, due to increased aggressiveness and resistance to medical treatment (38). Present data confirm our recent findings of significantly worse prognosis of c.35 G>A *KRAS* mutant pts treated with first line FIr-B/FOx (39), even in a small cohort of MCRC patients. Here we report for the first time the c.35 G>A *KRAS* mutant genotype as prognostic biomarker of unfavourable clinical outcome, significantly related to worse efficacy (PFS) of second line treatments. Further prospective studies will confirm prognostic and predictive value of c.35 G>A *KRAS* mutation in MCRC patients.

In conclusion, clinical outcome of MCRC progressing to first line FIr-B/FOx regimen may be significantly favourable in pts re-challenging triplet chemotherapy associated with targeted agent compared to other second line treatments and significantly worse in c.35 G>A mutant compared to wild-type and other mutant *KRAS* patients.

## Figures and Tables

**Table I.** Clinical management of MCRC patients after progression to first-line FIr-B/FOx regimen.

	Overall	*KRAS* genotype	
	No. of patients (%)	Wild-type (%)	Mutant (%)
Total no.	54	26	25
Second line treatment	40 (74.1)	21 (80.8)	17 (68)
Medical treatment	36 (66.7)	18 (69.2)	16 (64)
Triplet chemotherapy plus targeted agent	10 (18.5)	5 (19.2)	5 (20)
Triplet chemotherapy plus bevacizumab	7	3	4
Triplet chemotherapy plus cetuximab	3	2	1
Triplet regimen	19 (35.2)	10 (38.5)	7 (28)
Doublet chemotherapy plus bevacizumab	5	1	4
Doublet chemotherapy plus cetuximab	13	8	3
Triplet chemotherapy	1	1	-
Doublet regimen	3 (5.6)	2 (7.6)	1 (4)
Mono-chemotherapy plus bevacizumab	3	2	1
Mono therapy	4 (7.4)	1 (3.8)	3 (12)
Mono-chemotherapy	3	-	3
Panitumumab	1	1	-
Surgery	4 (7.4)	3 (11.5)	1 (4)
Death events without further treatment	14 (25.9)	5 (19.2)	8 (32)

**Table II.** Features of second line treated patients according to *KRAS* genotype.

	Overall treated	*KRAS* wild-type	*KRAS* mutant
	Total no. (%)	Total no. (%)	Total no. (%)
No. of patients	40	21 (55.3)	17 (44.7)
Sex			
Male/female	26/14	17/4	8/9
Age, years			
Median	65	64	66
Range	46-74	46-73	51-74
≥65 years	18 (45)	9 (43)	8 (47)
Metastatic disease			
Metachronous	15 (37.5)	10 (48)	5 (29)
Synchronous	25 (62.5)	11 (52)	12 (71)
Primary tumor			
Colon	18 (45)	6 (29)	11 (65)
Rectum	22 (55)	15 (71)	6 (35)
Sites of metastases			
Liver	22 (55)	10 (48)	11 (65)
Lung	17 (42.5)	10 (48)	5 (29)
Lymph nodes	17 (42.5)	10 (48)	5 (29)
Local	13 (32.5)	9 (43)	4 (23)
Other	12 (30)	6 (29)	5 (29)
No. of involved sites			
1	16 (40)	9 (43)	8 (47)
≥2	24 (60)	12 (57)	9 (53)
Single metastatic sites			
Liver-limited	9 (22.5)	3 (14)	6 (35)
Other than liver	7 (17.5)	6 (29)	2 (12)
Lung	4 (10)	4 (19)	1 (6)
Lymph nodes	2 (5)	2 (9)	-
Local	-	-	-
Other	1 (2.5)	-	-
Multiple metastatic sites	24 (60)	12 (57)	9 (53)
Liver metastases			
Single	3 (7.5)	1 (5)	2 (12)
Multiple	20 (50)	9 (43)	9 (53)
Previous adjuvant chemotherapy	7 (17.5)	5 (24)	1 (6)
FA/5-FU bolus	4 (10)	3 (14)	-
Capecitabine	-	-	-
FOLFOX4	3 (7.5)	2 (9)	1 (6)
Previous radiotherapy	5 (12.5)	4 (19)	1 (6)
RT alone	1 (2.5)	1 (5)	-
RT+CT (5-FU continous infusion)	2 (5)	2 (9)	-
RT+CT (XELOX)	2 (5)	1 (5)	1 (6)

**Table III.** Prognostic relevance.

A, Activity, efficacy and effectiveness of second line after FIr-B/FOx regimen according to *KRAS* genotype						
	All		*KRAS* wild-type		*KRAS* mutant	
	Intent-to-treat analysis		Intent-to-treat analysis		Intent-to-treat analysis	
	No	%	No	%	No	%
Enrolled patients	40	100	21	100	17	100
Evaluable patients	34	89	18	86	14	93
Objective response	13	38 (CI ± 17)	9	50 (CI ± 24)	4	29 (CI ± 25)
Partial response	10	29	7	39	3	21
Complete response	3	9	2	11	1	7
Stable disease	10	29	5	28	3	21
Progressive disease	11	32	3	17	7	50
Median PFS, months	10		10		10	
Range	1–32+		3–31+		1–32+	
Progression events	33	82.5	17	81	14	82
Median OS, months	14		17		12	
Range	1–51+		5+−51+		1–39+	
Deaths	26	65	13	62	11	65
Metastasectomies	5	12.5	3	15	2	12
Peritoneal carcinomatosis	2		1		1	
Liver	2		1		1	
Lymph nodes	1		1		-	
Pathologic complete responses	1	20	-	-	1	50
PFS, progression-free survival; OS, overall survival.						

B, Activity, efficacy and effectiveness of second line intensive treatments				
	Intent-to-treat analysis			
	Triplet chemotherapy plus targeted agent		Triplet regimen	
	No.	%	No.	%
Enrolled patients	10	100	19	100
Evaluable patients	10	100	18	95
Objective response	8	80 (CI ± 26)	5	28 (CI ± 21)
Partial response	5	50	5	28
Complete response	3	30	-	-
Stable disease	1	10	6	33
Progressive disease	1	10	7	39
Median PFS, months	13		8	
Range	4–32+		1+−17	
Progression events	6	60	17	89
Median OS, months	NR		11	
Range	6+−39+		1+−38	
Deaths	2	20	16	84
Metastasectomies	4	40	1	6
Peritoneal	1		1	
Liver	2		-	
Lymph nodes	1		-	
Pathologic complete responses	1	25	-	-
PFS, progression-free survival; OS, overall survival. NR, not reached.				
